# Maternal Fatty Acids and Their Association with Birth Outcome: A Prospective Study

**DOI:** 10.1371/journal.pone.0147359

**Published:** 2016-01-27

**Authors:** Akshaya Meher, Karuna Randhir, Savita Mehendale, Girija Wagh, Sadhana Joshi

**Affiliations:** 1 Department of Nutritional Medicine, Interactive Research School for Health Affairs, Bharati Vidyapeeth University, Pune, 411043, India; 2 Dept of Obstetrics and Gynaecology, Bharati Medical College and Hospital, Bharati Vidyapeeth University, Pune, 411043, India; University of Naples Federico II, ITALY

## Abstract

Maternal nutrition, especially LCPUFA, is an important factor in determining fetal growth and development. Our earlier cross sectional study reports lower docosahexanoic acid (DHA) levels at the time of delivery in mothers delivering low birth weight (LBW) babies. This study was undertaken to examine the role of the maternal omega-3 and omega-6 fatty acid profile across the gestation in fetal growth. This is a hospital based study where women were recruited in early gestation. Maternal blood was collected at 3 time points, i.e., T1 = 16^th^–20^th^ week, T2 = 26^th^–30^th^ week and T3 = at delivery. Cord blood was collected at delivery. At delivery, these women were divided into 2 groups: those delivering at term a baby weighing >2.5kg [Normal birth weight (NBW) group] and those delivering at term a baby weighing <2.5kg [LBW group]. The study reports data on 111 women recruited at T1, out of which 60 women delivered an NBW baby at term and 51 women delivered an LBW baby at term. Fatty acids were analysed using gas chromatography. At T1 of gestation, maternal erythrocyte DHA levels were positively (p<0.05) associated with baby weight. Maternal plasma and erythrocyte arachidonic acid and total erythrocyte omega-6 fatty acid levels at T2 were higher (p<0.05 for both) in the LBW group. Total erythrocyte omega-3 fatty acid levels were lower (p<0.05) while total erythrocyte omega-6 fatty acid levels were higher (p<0.05) in the LBW group at delivery. Our data demonstrates the possible role of LCPUFA in the etiology of LBW babies right from early pregnancy.

## Introduction

Low birth weight (LBW) is associated with higher mortality, morbidity, disability in infancy and childhood and also has a long-term impact on health outcomes in adult life [1]. Globally 20 million LBW infants are born every year; out of which, 96.5% of them are from developing countries [2]. This is of significance for India, which has recently been referred to as the world capital for LBW babies [3].

Maternal nutritional status is well known to be an important determinant of placental and fetal growth [4]. Among the different nutrients, long chain poly unsaturated fatty acids (LCPUFA) like docosahexaenoic acid (DHA) and arachidonic acid (ARA) are vital during pregnancy for the fetus since they form structural constituents of the membrane lipids of the developing brain and central nervous system [5]. The developing fetus completely depends on the maternal essential fatty acid supply, and a maternal shortage could result in an adverse pregnancy outcome [6]. A number of cross sectional human studies carried out in our department have extensively demonstrated lower LCPUFA levels in pregnancy complications like preeclampsia [7–9].

Further, in normotensive pregnancies we and others have also reported lower maternal LCPUFA levels at the time of delivery [10–15]. However, since these studies were carried out at the end of pregnancy; it is not clear whether the differences in the LCPUFA proportions were present early in pregnancy.

There are limited studies which report the plasma fatty acid profile of women only in early pregnancy and their association with birth weight [6, 16–17]. However; it is likely that the results may be influenced by changes in fatty acid status in late gestation. Similarly other studies have examined only maternal erythrocyte fatty acid levels across the gestation [18] or only maternal plasma fatty acid levels across the gestation [19]. Reports indicate that, the amounts of umbilical cord fatty acids are correlated with the amounts in maternal blood, and are critical for later health outcomes of children [20]. Thus, it is vital to examine the association of maternal fatty acids with cord fatty acids for better understanding of the effects of maternal LCPUFA levels leading to LBW babies.

The objective of the current study was to examine the potential role of the maternal omega-3 and omega-6 fatty acid profile in fetal growth by investigating the association between maternal concentrations of these fatty acids across the gestation and birth outcome measures (birth weight, birth length, head circumference and chest circumference at birth). Further, we prospectively compare the levels of different LCPUFA in both plasma and erythrocyte in maternal and umbilical cord blood samples of mothers delivering normal birth weight baby (NBW) (>2.5kg) and mothers delivering LBW baby.

## Material and Methods

### Setting

Pregnant women were enrolled for this longitudinal study from Department of Obstetrics and Gynaecology, Bharati hospital Pune, India. The study was approved by the Bharati Vidyapeeth Medical College Institutional Ethical Committee (Ref No.: BVDU/MC/02) and written consent was taken from each subject at the time of enrollment. The current study is a part of ongoing prospective departmental study which recruits all healthy women at 16^th^-20^th^ week of gestation and follows them throughout pregnancy. Subjects included in this study were only those women with singleton pregnancy, delivering at term (total gestation ≥ 37 weeks) and having no medical or obstetrical complications. At delivery, these women were divided into 2 groups; those delivering at term a baby weighing >2.5kg [Normal birth weight (NBW) group] and those delivering at term a baby weighing <2.5kg [Low birth weight (LBW) group]. Pregnant women with medical problems like multiple gestation, chronic hypertension, type I or type II diabetes mellitus, seizure disorder, alcohol or drug abuse, preeclampsia, gestational diabetes, renal or liver disease and anemia were excluded from the study.

**Fig 1:**
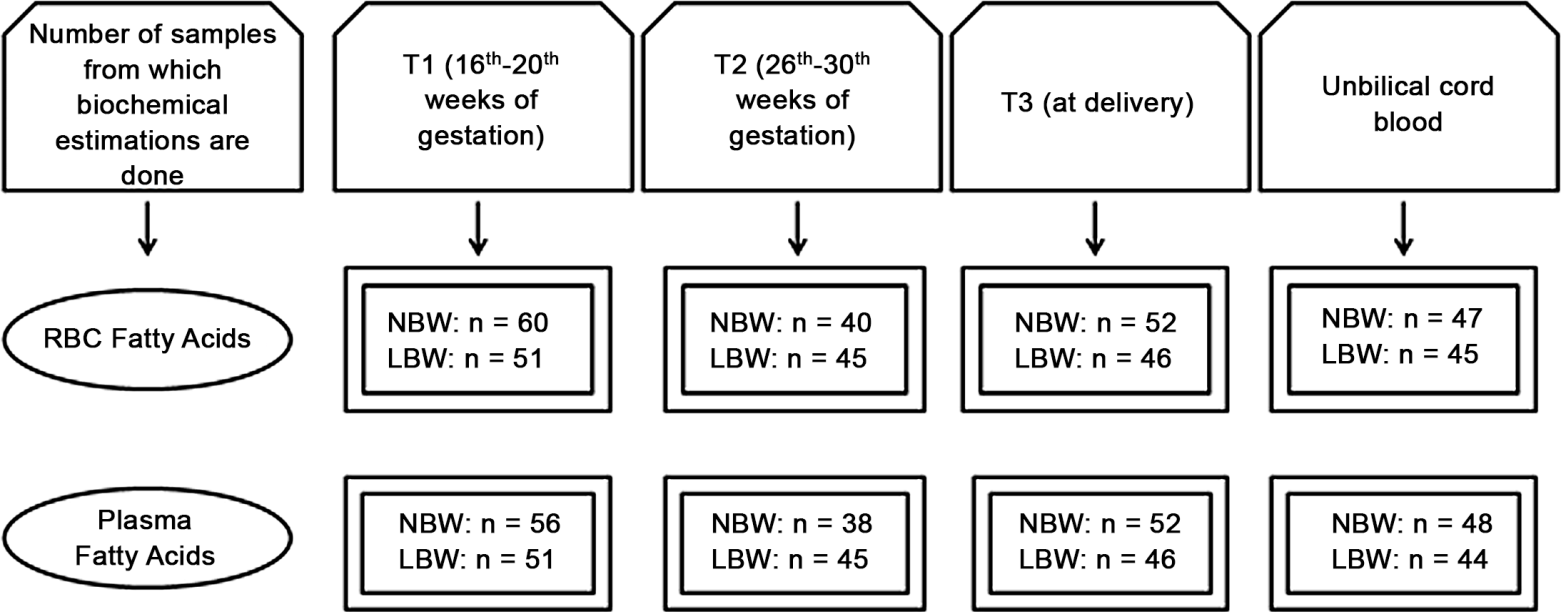
Flow chart showing number of maternal and cord plasma and erythrocyte samples analyzed for different parameters at various time points: T1 = 16^th^–20^th^ weeks, T2 = 26^th^–30^th^ week, T3 = at delivery and cord samples.

The first sample was obtained between 16^th^-20^th^ weeks of gestation (T1), the second between 26^th^-30^th^ weeks of gestation (T2) and the third sample was taken just before going to the labor room (T3). Umbilical cord blood was also collected. Study reports data on 111 women recruited at T1, out of which 60 women delivered a NBW baby at term and 51 women delivered a LBW baby at term (Fig 1). The gestational age in the current study was determined by last menstrual period and ultrasound examination.

### Sociodemographic and anthropometric measures

Information on age, obstetric history, family background and socioeconomic status was collected.

### Dietary assessments

Pregnant women were administered with a food frequency questionnaire during T1, T2 and at delivery to estimate the frequency of consumption of foods rich in omega-3 fatty acids. All pregnant women had to indicate the frequency of each food consumed during the last one month for which scores were calculated. For example, an item consumed once a week has a score of 4 while that consumed daily has a score of 30. These foods were identified using “Nutritive Values of Indian Foods” [21]. The food frequency questionnaire has been used by the department in a number of studies on pregnant women [22–24].

### Sample collection and processing

At each visit fasting blood (10mL) was collected from the subjects into ethylene di amine tetra acetic acid (EDTA) tubes. All blood samples were immediately layered on histopaque (Sigma-Aldrich, St Louis, MO, USA) and centrifuged at 2000rpm for 30min to separate the plasma and erythrocytes. The erythrocyte fraction was washed 3 times with normal saline. Plasma and erythrocytes were stored at -80°C until further analysis.

### Fetal growth measures

Birth weight, baby length, baby head circumference and chest circumference were recorded. Birth weight was recorded using a digital weighing scale (Zeal medical private limited, India) with an accuracy of 10gm. The length was measured to the nearest 0.1cm using a portable infantometer. The head circumference and chest circumference was measured using a fiber glass measuring tape which was placed around the head, just above the eyebrows anteriorly, and around the most prominent bulge posteriorly. The chest circumference was measured using fiber glass measuring tape which was placed around the lower chest. These procedures have been described in our earlier study [25].

### Plasma and erythrocyte fatty acid analysis

LCPUFA were analyzed from the plasma and erythrocyte samples using gas chromatography and the method has been described by us earlier [7–9, 24, 26–28]. Briefly, transesterification of the total plasma and erythrocyte fatty acids were performed using hydrochloric acid–methanol. Methyl esters were separated using a PerkinElmer gas chromatograph (SP 2330, 30-m capillary Supelco column; Perkin Elmer, Shelton, CT, USA). Peaks were identified by comparison with standard fatty acid methyl esters (Sigma-Aldrich). Fatty acids were expressed as g per 100 g fatty acid. The saturated fatty acids (SFAs) include myristic acid (Myr), palmitic acid (Pal) and stearic acid (Ste), while the monounsaturated fatty acids (MUFAs) include myristoleic acid (Myro), palmitoleic acid (Palo), oleic acid (Ole) and nervonic acid (NA). The omega-3 fatty acids included alpha linolenic acid (ALA), eicosapentanoic acid (EPA) and DHA, while omega-6 fatty acids included linoleic acid (LA), gamma linolenic acid (GLA), di-homo-gamma linolenic acid (DGLA), docosapentaenoic acid (DPA) and ARA.

### Statistical analysis

Values are reported as mean ± S.D. The data were analyzed using the SPSS/PC+ package (Version 20.0, Chicago, IL, USA). The data was checked for normal distribution by testing for skewness and kurtosis. Skewed variables were log to the base 10 transformed. Mean values of the various parameters were compared using independent t test for comparison between NBW and LBW group. Mean values of the various parameters were compared using one way analysis of variance (ANOVA) and the Fishers post-hoc least significant difference (LSD) test for comparison within the NBW and LBW group. Correlation between these variables was studied using Pearson’s correlation analysis after adjusting for age, body mass index (BMI) and gestational age at the time of blood sampling. Chi-square test was used for comparison of categorical variables. To compare two proportions, Z test of proportions was used.

The variable sample number (n) in different measures was either due to loss of follow up at various time points across gestation or insufficient sample volume available. Results corresponding to p-values lower than 0.05 (5%) are described as significant and reported. Statistical analysis was carried out on two sets of data. First set of data includes all the women who have participated in the study. Second set of data was analyzed in women from whom the parameters were analyzed for all time points as well as in cord. The results of the first set of data have been shown in the tables. However, similar results and trends were observed for the second set of data (data not shown).

## Results

### Maternal and neonatal characteristics

Table 1 shows the demographic characteristics of normotensive mothers and their neonates. Maternal BMI at T1 and T3 was lower (p<0.05 for both) in the LBW group as compared to NBW group. All the neonatal characteristics including baby weight, baby length, head circumference and chest circumference were lower (p<0.01 for all) in the LBW group as compared to NBW group.

**Table 1:** Maternal and Neonatal Characteristics.

Maternal characteristics (Mean ± SD)		
	NBW (n = 60)	LBW (n = 51)
**Age (yr)**	23.82 ± 3.92	22.80 ± 3.19
**Income (INR)**	13223 ± 13703	8294 ± 6623*
**BMI (Kg/m2)**		
**T1**	21.79 ± 3.55	20.40 ± 3.16*
**T2**	23.71 ± 3.69	22.25 ± 2.97
**T3**	25.40 ± 3.70	23.53 ± 3.21*
**Gestation (wks)**		
**T1**	19.69 ± 2.59	19.09 ± 2.20
**T2**	29.67 ± 2.61	29.03 ± 2.11
**T3**	39.00 ± 1.01	38.51 ± 1.44
**Sys BP (mmHg)**		
**T1**	115.82 ± 8.75	116.6 ± 7.60
**T2**	116.76 ± 7.83	117.49 ± 5.89
**T3**	119.12 ± 7.53	120.87 ± 8.39
**Dias BP (mmHg)**		
**T1**	74.25 ± 5.31	73.15 ± 6.86
**T2**	73.51 ± 6.33	75.74 ± 6.09
**T3**	76.96 ± 6.14	76.78 ± 4.78
**Women delivering NBW and LBW baby (%)**	54	45
	**Neonatal characteristics**	
**Baby weight (kg)**	2.97 ± 0.29	2.31 ± 0.17**
**Baby length (cm)**	48.89 ± 2.81	46.01 ± 2.73**
**Baby HC (cm)**	33.99 ± 1.40	32.35 ± 1.84 **
**Baby CC (cm)**	32.43 ± 1.74	30.68 ± 1.77**

NBW—normal birth weight; LBW—low birth weight; BMI—body mass index; Sys BP—Systolic blood pressure; Dias BP—diastolic blood pressure; HC—head circumference; CC—chest circumference; T1 = 16th–20th week, T2 = 26th–30th week, T3 = at delivery.

*p<0.05

**p<0.01 as compared with NBW group.

### Association of maternal and cord fatty acids with birth outcome

At T1, the maternal erythrocyte fatty acids like SFA, MUFA, total omega-3 fatty acids, and ARA were not associated with any of the birth outcome parameters. In contrast, maternal erythrocyte DHA levels were positively associated with baby weight (n = 105, r = 0.222, p = 0.025). Further, there was positive association between maternal total erythrocyte omega-6 fatty acid with baby length (n = 97, r = 0.223, p = 0.031). There was no association between maternal plasma fatty acids like SFA, MUFA, total omega-3 fatty acids, total omega-6 fatty acids, DHA and ARA with any of the birth outcome parameters.

At T2, the maternal erythrocyte fatty acids like SFA, MUFA, total omega-3 fatty acids, total omega-6 fatty acids, DHA, and ARA were not associated with any of the birth outcome parameters. Maternal plasma SFA levels were positively associated with baby head circumference (n = 69, r = 0.257, p = 0.037). There was a negative association between maternal plasma omega-6 fatty acids with baby weight (n = 78, r = -0.255, p = 0.027) and baby head circumference (n = 69, r = -0.267, p = 0.030).

At T3, the maternal erythrocyte fatty acids like SFA, total omega-3 fatty acids, and ARA were not associated with any of the birth outcome parameters. There was a negative association between MUFA levels (n = 87, r = -0.288, p = 0.008), total omega-6 fatty acids (n = 87, r = -0.388, p = 0.000) and positive association between DHA levels (n = 87, r = 0.241, p = 0.027) with baby head circumference. Maternal plasma total omega-6 fatty acid levels were negatively associated (n = 86, r = -0.304, p = 0.005) and ARA levels were positively associated (n = 86, r = 0.262, p = 0.071) with baby chest circumference.

Cord erythrocyte and plasma SFA, MUFA, total omega-3 fatty acids, total omega-6 fatty acids, DHA, and ARA levels were not associated with any of the birth outcome parameters.

### Associations between maternal erythrocyte fatty acids at T1, T2, T3 and cord erythrocyte fatty acids

The SFA levels in the cord were not associated with maternal SFA levels at any time point during gestation. Cord MUFA and total omega-3 fatty acid levels were positively associated with maternal MUFA (n = 87, r = 0.284, p = 0.008) and total omega-3 fatty acid (n = 87, r = 0.392, p = 0.000) at T1 respectively. Cord total omega-6 fatty acid levels were not associated with maternal total omega-6 fatty acid levels at any time point during gestation. Cord DHA was positively associated with maternal DHA at all the three time points (T1: n = 87, r = 0.493, p = 0.000; T2: n = 67, r = 0.565, p = 0.000; T3: n = 82, r = 0.243, p = 0.029). Cord ARA was negatively associated with maternal ARA at T1 and T3 (T1: n = 87, r = -0.229, p = 0.034; T3: n = 82, r = -0.428, p = 0.000) (Table 2).

**Table 2:** Associations between cord fatty acids and maternal fatty acids at T1, T2, T3 in whole cohort.

Fatty Acids (g/100g)	Whole Cohort					
	Erythrocyte			Plasma		
	n	r	p	n	r	p
***Cord DHA with Maternal DHA***						
T1 (16^th^-20^th^ week)	**87**	**0.493**	**0.000**	**86**	**0.396**	**0.000**
T2 (26^th^-30^th^ week)	**67**	**0.565**	**0.000**	**67**	**0.456**	**0.000**
T3 (at delivery)	**82**	**0.243**	**0.029**	**82**	**0.335**	**0.002**
***Cord ARA with Maternal ARA***						
T1 (16^th^-20^th^ week)	**87**	**-0.229**	**0.034**	86	0.009	0.934
T2 (26^th^-30^th^ week)	67	-0.227	0.066	67	0.121	0.335
T3 (at delivery)	**82**	**-0.428**	**0.000**	**82**	**-0.584**	**0.000**
***Cord Total Omega-3 Fatty Acids with Maternal Total Omega-3 Fatty Acids***						
T1 (16^th^-20^th^ week)	**87**	**0.392**	**0.000**	**86**	**0.422**	**0.000**
T2 (26^th^-30^th^ week)	67	0.200	0.108	**67**	**0.336**	**0.006**
T3 (at delivery)	82	0.203	0.070	**82**	**0.322**	**0.003**
***Cord Total Omega-6 Fatty Acids withMaternal Total Omega-6 Fatty Acids***						
T1 (16^th^-20^th^ week)	87	-0.178	0.102	86	0.159	0.147
T2 (26^th^-30^th^ week)	67	0.019	0.878	67	0.096	0.441
T3 (at delivery)	82	-0.091	0.421	**82**	**-0.294**	**0.008**
***Cord SFA with Maternal SFA***						
T1 (16^th^-20^th^ week)	87	0.078	0.477	86	0.174	0.112
T2 (26^th^-30^th^ week)	67	0.115	0.359	67	0.027	0.828
T3 (at delivery)	82	0.176	0.115	82	-0.113	0.316
***Cord MUFA with Maternal MUFA***						
T1 (16^th^-20^th^ week)	**87**	**0.284**	**0.008**	86	0.172	0.115
T2 (26^th^-30^th^ week)	67	0.238	0.054	**67**	**0.288**	**0.019**
T3 (at delivery)	82	0.032	0.779	**82**	**0.276**	**0.013**

T1 = 16th–20th week, T2 = 26th–30th week, T3 = at delivery.

ARA–arachidonic acid, DHA–docosahexaenoic acid, total omega-3: (alpha linolenic acid + eicosapentaenoic acid + docosahexaenoic acid), total omega-6: (linoleic acid + gamma linolenic acid + di homo gamma linolenic acid + arachidonic acid + docosapentaenoic acid), SFA- saturated fatty acids, MUFA- mono unsaturated fatty acids. For Pearson's correlation coefficients, p<0.05.

### Associations between maternal plasma fatty acids at T1, T2, T3 and cord plasma fatty acids

Cord SFA levels were not associated with maternal SFA levels at any time point during gestation. Cord MUFA levels were positively associated with maternal MUFA at T2 and T3 (T2: n = 67, r = 0.288, p = 0.019; T3: n = 82, r = 0.276, p = 0.013). Cord total omega-3 fatty acids were positively associated with maternal total omega-3 fatty acids at all three time points during gestation (T1: n = 86, r = 0.422, p = 0.000; T2: n = 67, r = 0.336, p = 0.006; T3: n = 82, r = 0.322, p = 0.003). Cord total omega-6 fatty acids was negatively associated with maternal total omega-6 fatty acids at T3 (n = 82, r = -0.294, p = 0.008). Cord DHA was positively associated with maternal DHA at all the three time points (T1: n = 86, r = 0.396, p = 0.000; T2: n = 67, r = 0.456, p = 0.000; T3: n = 82, r = 0.335, p = 0.002). Cord ARA was negatively associated with maternal ARA at T3 (n = 82, r = -0.584, p = 0.000) (Table 2).

### Maternal and cord erythrocyte fatty acids levels in different groups at different gestational time points

At T1, there was no change in the levels of SFA, MUFA, total omega-3 and omega-6 fatty acids, and ARA between the two groups. There was a trend towards reduction in the levels of DHA in the LBW group as compared to NBW group, although it was not statistically significant (Table 3).

**Table 3:** Changes in erythrocyte fatty acid proportions from 16^th^ week of gestation till delivery (T1 = 16^th^–20^th^ week, T2 = 26^th^–30^th^ week, T3 = at delivery, Cord erythrocytes).

	NBW				LBW			
	T1 (n=60)	T2 (n=40)	T3 (n=52)	Cord Erythrocytes (n=47)	T1 (n=51)	T2 (n=45)	T3 (n=46)	Cord Erythrocytes (n=45)
**Myr**	0.32 ± 0.12	0.31 ± 0.12	0.34 ± 0.17	0.40 ± 0.38^**$**^	0.31 ± 0.13	0.29 ± 0.09	0.33 ± 0.13	0.32 ± 0.09
**Myro**	0.07 ± 0.11	0.07 ± 0.10	0.08 ± 0.14	0.06 ± 0.07	0.07 ± 0.16	0.05 ± 0.08	0.04 ± 0.08	0.09 ± 0.14
**Pal**	22.33 ± 1.62	22.36 ± 1.66	23.44 ± 3.75^**@$**^	24.16 ± 2.00^**@$**^	22.33 ± 1.99	22.78 ± 1.30	24.89 ± 2.24^*@$^	24.57 ± 2.52^**@$**^
**Palo**	0.19 ± 0.10	0.21 ± 0.12	0.30 ± 0.18^**@$**^	0.38 ± 0.15^**@$#**^	0.21 ± 0.19	0.21 ± 0.13	0.32 ± 0.31^**@$**^	0.37 ± 0.17^**@$**^
**Ste**	14.98 ± 0.95	14.79 ± 0.72	15.04 ± 1.38	16.71 ± 1.62^**@$#**^	15.49 ± 1.40^*^	15.12 ± 1.49	15.85 ± 2.23^*$^	16.95 ± 1.63^**@$#**^
**Ole**	9.37 ± 0.90	9.33 ± 0.74	9.63 ± 2.10	7.60 ± 1.33^**@$#**^	9.11 ± 1.02	8.93 ± 1.09^*^	9.48 ± 1.47^**$**^	7.65 ± 1.21^**@$#**^
**LA**	10.75 ± 1.42	10.66 ± 1.52	9.80 ± 3.41^**@**^	4.69 ± 2.38^**@$#**^	10.79 ± 1.20	11.11 ± 2.43	10.70 ± 4.07	4.65 ± 2.30^**@$#**^
**GLA**	0.11 ± 0.10	0.11 ± 0.24	0.07 ± 0.07	0.07 ± 0.06	0.07 ± 0.10^*^	0.05 ± 0.04	0.10 ± 0.10^**$**^	0.09 ± 0.11^**$**^
**ALA**	0.23 ± 0.13	0.23 ± 0.15	0.24 ± 0.15	0.38 ± 0.11^**@$#**^	0.24 ± 0.10	0.29 ± 0.22	0.25 ± 0.12	0.38 ± 0.11^**@$#**^
**DGLA**	1.63 ± 0.38	1.68 ± 0.32	1.65 ± 0.47	2.19 ± 0.63^**@$#**^	1.54 ± 0.40	1.65 ± 0.37	1.63 ± 0.42	2.28 ± 1.20^**@$#**^
**ARA**	13.27 ± 1.19	12.81 ± 1.07	12.12 ± 2.43^**@**^	15.48 ± 1.81^**@$#**^	13.82 ± 2.14	13.48 ± 1.45^*^	12.59 ± 2.75^**@**^	15.48 ± 2.33^**@$#**^
**EPA**	0.50 ± 0.79	0.60 ± 0.73	0.76 ± 0.92	0.53 ± 0.69	0.62 ± 0.84	0.65 ± 0.86	0.51 ± 0.90^*^	0.54 ± 0.89
**NA**	0.92 ± 0.44	1.11 ± 0.43	1.25 ± 0.72^**@**^	2.10 ± 0.71^**@$#**^	0.95 ± 0.30	1.20 ± 0.45^**@**^	1.27 ± 0.66^**@**^	1.96 ± 0.56^**@$#**^
**DPA**	0.82 ± 0.28	0.79 ± 0.34	0.65 ± 0.35^**@$**^	0.31 ± 0.29^**@$#**^	0.72 ± 0.25 ^*^	0.69 ± 0.26	0.58 ± 0.27^**@$**^	0.30 ± 0.20^**@$#**^
**DHA**	2.65 ± 0.85	2.67 ± 0.80	2.55 ± 0.87	3.03 ± 0.89^**@$#**^	2.41 ± 0.63	2.69 ± 0.77	2.38 ± 0.82	3.01 ± 1.05^**@#**^
**N3**	3.38 ± 1.04	3.50 ± 1.01	3.55 ± 1.09	3.94 ± 1.02^**@**^	3.27 ± 0.94	3.63 ± 1.15	3.15 ± 1.15^*$^	3.93 ± 1.23^**@#**^
**N6**	26.58 ± 1.87	26.05 ± 1.44	24.29 ± 3.16^**@$**^	22.74 ± 2.36^**@$#**^	26.95 ± 2.59	26.99 ± 2.13^*^	25.59 ± 3.34^*@$^	22.81 ± 2.54^**@$#**^
**SFA**	37.63 ± 2.05	37.46 ± 2.03	38.83 ± 4.42^**@$**^	41.27 ± 3.19^**@$#**^	38.13 ± 3.27	38.19 ± 2.30	41.07 ± 3.54^**@$^	41.85 ± 3.96^**@$**^
**MUFA**	10.55 ± 1.02	10.72 ± 0.94	11.26 ± 1.93^**@**^	10.13 ± 1.43^**#**^	10.33 ± 1.17	10.39 ± 1.20	11.11 ± 1.49^**@$**^	10.07 ± 1.18^**#**^

NBW—normal birth weight; LBW—low birth weight; T1 = 16th–20th week, T2 = 26th–30th week, T3 = at delivery.

**p<0.01, *p<0.05 as compared with NBW at the corresponding time points; @@p<0.01, @p<0.05 as compared with T1, $p<0.05 as compared with T2; ##p<0.01, #p<0.05 as compared with T3. Myr–Myristic acid, Myro–Myristoleic acid, Pal—palmitic acid, Palo—palmitoleic acid, Ste–Stearic acid, Ole—oleic acid, LA—linoleic acid, GLA—gamma linolenic acid, ALA—alpha linolenic acid, DGLA–di homo gamma linolenic acid, ARA—arachidonic acid, EPA—eicosapentaenoic acid, NA–Nervonic acid, DPA,omega-6—Docosapentaenoic acid, DHA—docosahexaenoic acid, saturated fatty acids (SFA): (Myristic acid + palmitic acid + stearic acid), monounsaturated fatty acids (MUFA): (Myristoleic acid + palmitoleic acid + oleic acid + nervonic acid), total omega-3: (alpha linolenic acid +eicosapentaenoic acid + docosahexaenoic acid), total omega-6: (linoleic acid + gamma linolenic acid + dihomo gamma linolenic acid + arachidonic acid + docosapentaenoic acid.

At T2, the levels of SFA, MUFA, total omega-3 fatty acids, and DHA were similar between the two groups. However, the total omega-6 fatty acids and ARA levels at T2 were higher (p<0.05 for both) in the LBW group as compared to NBW group (Table 3).

At the time of delivery (T3), SFA levels were higher (p<0.01) in the LBW group as compared to NBW group although the levels of MUFA were similar between the two groups. The total omega-3 fatty acid levels were lower while the total omega-6 fatty acid levels were higher (p<0.05 for both) in the LBW group as compared to NBW group. However, there was no change in the levels of DHA and ARA in between the two groups (Table 3).

The levels of SFA, MUFA, total omega-3 and omega-6 fatty acids, DHA and ARA in the cord were similar between the two groups (Table 3).

### Changes in erythrocyte fatty acid levels from 16^th^ week of gestation till delivery and in the cord in NBW and LBW groups

In both NBW and LBW groups, there was no difference in the levels of SFA, MUFA, total omega-3 fatty acids, total omega-6 fatty acids, DHA, and ARA at T2 as compared with T1.

The levels of SFA and MUFA were higher (p<0.05 for both) while the levels of total omega-6 fatty acids and ARA were lower (p<0.05 for both) at the time of delivery (T3) as compared with T1. There was no difference in the levels of omega-3 fatty acids and DHA at T3 as compared with T1 (Table 3).

In the NBW group, the levels of SFA were higher (p<0.05) while that of total omega-6 fatty acids and ARA were lower (p<0.05) at T3 as compared with T2. Similarly, in the LBW group, the levels of MUFA were higher (p<0.05) and total omega-6 fatty acids, total omega-3 fatty acids were lower (p<0.05 for both) at T3 as compared with T2. There was no change in the levels of DHA at T3 as compared with T2 in both the groups (Table 3).

In the NBW group, the cord levels of SFA, DHA and ARA were higher and total omega-6 fatty acids were lower(p<0.05 for all) as compared with maternal levels at all the time points. The cord MUFA levels were lower (p<0.05) as compared with maternal MUFA levels at delivery. Cord total omega-3 fatty acid levels were higher as compared to maternal levels at T1 (Table 3).

In the LBW group, the cord levels of SFA were higher (p<0.05 for both) as compared with maternal levels at T1, T2.The cord MUFA levels were lower (p<0.05) as compared with maternal MUFA levels at delivery. Cord total omega-3 fatty acid, DHA levels were higher (p<0.05 for both) as compared with maternal levels at T1, T3 in the LBW group. The cord levels of total omega-6 fatty acid were lower and ARA were higher (p<0.05 for both) as compared with maternal levels at all the time points (Table 3).

### Maternal and cord plasma fatty acids levels in different groups at different gestational time points

There was no change in the levels of SFA, MUFA, total omega-3 and omega-6 fatty acids and DHA between the two groups across the gestation. In contrast, maternal ARA levels at T2 was higher (p<0.05) in the LBW group as compared to NBW group (Table 4).

**Table 4:** Changes in plasma fatty acid proportions from 16^th^ week of gestation till delivery (T1 = 16^th^–20^th^ week, T2 = 26^th^–30^th^ week, T3 = at delivery, cord plasma).

	NBW				LBW			
	T1 (n=56)	T2 (n=38)	T3 (n=52)	Cord Plasma (n=48)	T1 (n=51)	T2 (n=45)	T3 (n=46)	Cord Plasma (n=44)
**Myr**	1.03 ± 0.43	1.12 ± 0.46	0.99 ± 0.42	0.78 ± 0.24^**@$#**^	0.91 ± 0.46	1.01 ± 0.40	0.87 ± 0.37	0.84 ± 0.44
**Myro**	0.07 ± 0.06	0.08 ± 0.05	0.06 ± 0.05	0.09 ± 0.09^**@#**^	0.08 ± 0.10	0.06 ± 0.06^*^	0.09 ± 0.12	0.09 ± 0.12
**Pal**	25.31 ± 2.68	26.73 ± 2.34^**@**^	27.42 ± 2.52^**@**^	27.76 ± 1.37^**@$**^	24.33 ± 2.30 ^*^	26.32 ± 2.25^**@**^	26.72 ± 2.34^**@**^	27.43 ± 2.76^**@$**^
**Palo**	1.28 ± 0.68	1.61 ± 0.82	2.00 ± 0.88^**@$**^	2.97 ± 1.03^**@$#**^	1.03 ± 0.60 ^*^	1.25 ± 0.54 ^*^	1.75 ± 0.87^**@$**^	2.76 ± 1.14^**@$#**^
**Ste**	6.03 ± 0.74	5.64 ± 0.71	6.14 ± 1.62	10.03 ± 2.04^**@$#**^	6.14 ± 0.74	5.60 ± 0.82	6.05 ± 1.78	9.77 ± 2.22^**@$#**^
**Ole**	14.65 ± 5.07	15.18 ± 1.80	15.61 ± 1.86	15.37 ± 1.63	14.35 ± 1.72	15.11 ± 2.00	15.63 ± 3.48^**@**^	15.11 ± 2.43
**LA**	34.55 ± 5.92	34.10 ± 3.86	30.79 ± 7.59^**@$**^	14.33 ± 7.13^**@$#**^	35.96 ± 3.78	35.35 ± 3.97	30.86 ± 7.55^**@$**^	15.83 ± 8.39^**@$#**^
**GLA**	0.20 ± 0.15	0.18 ± 0.13	0.15 ± 0.12	0.28 ± 0.17^**@$#**^	0.20 ± 0.18	0.16 ± 0.09	0.18 ± 0.11	0.24 ± 0.16^**$#**^
**ALA**	0.41 ± 0.29	0.42 ± 0.31	0.48 ± 0.26	0.33 ± 0.16^**#**^	0.43 ± 0.22	0.49 ± 0.28	0.44 ± 0.23	0.35 ± 0.14^**$**^
**DGLA**	1.53 ± 0.31	1.42 ± 0.27	1.49 ± 0.51	2.49 ± 0.68^**@$#**^	1.30 ± 0.54 ^**^	1.27 ± 0.43 ^*^	1.47 ± 0.51	2.23 ± 0.54 ^*@$#^
**ARA**	6.28 ± 1.26	5.35 ± 0.96^**@**^	6.22 ± 3.02	12.69 ± 2.79^**@$#**^	6.67 ± 1.34	5.85 ± 1.23 ^*^	6.58 ± 2.82	12.48 ± 3.48^**@$#**^
**EPA**	0.32 ± 0.51	0.34 ± 0.57	0.34 ± 0.59	0.34 ± 0.35	0.42 ± 0.48^*^	0.39 ± 0.53	0.54 ± 0.81	0.62 ± 0.78 ^*^
**NA**	0.50 ± 0.18	0.50 ± 0.18	0.61 ± 0.39	1.14 ± 0.39^**@$#**^	0.54 ± 0.20	0.50 ± 0.16	0.59 ± 0.30	1.02 ± 0.40^**@$#**^
**DPA**	0.16 ± 0.09	0.13 ± 0.07	0.13 ± 0.06	0.12 ± 0.08^**@**^	0.16 ± 0.07	0.16 ± 0.10	0.15 ± 0.10	0.12 ± 0.09^**@$**^
**DHA**	1.20 ± 0.38	1.03 ± 0.37	1.09 ± 0.44	1.70 ± 0.60^**@$#**^	1.12 ± 0.41	1.10 ± 0.37	1.10 ± 0.42	1.77 ± 0.84^**@$#**^
**N3**	1.93 ± 0.71	1.79 ± 0.82	1.90 ± 0.67	2.36 ± 0.72^**@$#**^	1.97 ± 0.71	1.99 ± 0.80	2.07 ± 1.02	2.74 ± 1.16^**@$#**^
**N6**	42.73 ± 5.94	41.18 ± 3.71	38.78 ± 5.01^**@$**^	29.91 ± 4.62^**@$#**^	44.30 ± 4.16	42.79 ± 4.31	39.23 ± 5.30^**@$**^	30.91 ± 5.36^**@$#**^
**SFA**	32.37 ± 3.11	33.49 ± 2.58	34.55 ± 3.09^**@**^	38.56 ± 2.15^**@$#**^	31.39 ± 2.65	32.93 ± 2.62^**@**^	33.65 ± 3.07^**@**^	38.04 ± 3.93^**@$#**^
**MUFA**	16.50 ± 5.16	17.36 ± 2.04	18.29 ± 2.40^**@**^	19.57 ± 1.98^**@$**^	16.00 ± 1.80	16.91 ± 2.06	18.05 ± 3.79^**@$**^	18.97 ± 3.00^**@$**^

NBW—normal birth weight; LBW—low birth weight; T1 = 16th–20th week, T2 = 26th–30th week, T3 = at delivery

**p<0.01, *p<0.05 as compared with NBW at the corresponding time points; @@p<0.01, @p<0.05 as compared with T1, $p<0.05 as compared with T2; ##p<0.01, #p<0.05 as compared with T3. Myr–Myristic acid, Myro–Myristoleic acid, Pal—palmitic acid, Palo—palmitoleic acid, Ste–Stearic acid, Ole—oleic acid, LA—linoleic acid, GLA—gamma linolenic acid, ALA—alpha linolenic acid, DGLA–di homo gamma linolenic acid, ARA—arachidonic acid, EPA—eicosapentaenoic acid, NA–Nervonic acid, DPA,omega-6—Docosapentaenoic acid, DHA—docosahexaenoic acid, saturated fatty acids (SFA): (Myristic acid + palmitic acid + stearic acid), monounsaturated fatty acids (MUFA): (Myristoleic acid + palmitoleic acid + oleic acid + nervonic acid), total omega-3: (alpha linolenic acid +eicosapentaenoic acid + docosahexaenoic acid), total omega-6: (linoleic acid + gamma linolenic acid + dihomo gamma linolenic acid + arachidonic acid + docosapentaenoic acid.

### Changes in plasma fatty acid levels from 16^th^ week of gestation till delivery and in the cord in NBW and LBW groups

In the NBW group, there was no change in the levels of SFA, MUFA, total omega-3 fatty acids, total omega-6 fatty acids, and DHA at T2 as compared with T1. The levels of ARA were lower (p<0.05) at T2 as compared with T1. In the LBW group, the levels of SFA were higher (p<0.05) at T2 as compared with T1. The levels of MUFA, total omega-3 fatty acids, total omega-6 fatty acids, DHA, and ARA were similar at T2 as compared with T1 (Table 4).

In both NBW and LBW group, the levels of SFA and MUFA were higher (p<0.05) while the levels of total omega-6 fatty acids were lower (p<0.05) at T3 as compared with T1. There was no change in the levels of total omega-3 fatty acids, DHA, and ARA at T3 as compared with T1 (Table 4).

In both NBW and LBW group, there was no change in the levels of SFA, total omega-3 fatty acids, DHA, and ARA at T3 as compared with T2. The levels of total omega-6 fatty acids were lower (p<0.05) at T3 as compared with T2. In the LBW group, the levels of MUFA were higher (p<0.05) at T3 as compared with T2 (Table 4).

In both NBW and LBW group, the cord levels of SFA, total omega-3 fatty acids, DHA, and ARA were higher (p<0.05 for all) as compared to maternal levels at all the time points across the gestation. The cord MUFA levels were higher (p<0.05 for both) as compared with maternal MUFA levels at T1 and T2. The cord total omega-6 fatty acid levels were lower (p<0.05 for all) as compared to maternal levels at all the time points across the gestation (Table 4).

### Frequency of Consumption of Omega-3 Fatty Acid Rich Foods

The frequency of consumption of omega-3 fatty acid rich foods was similar in both the groups at T1 (p = 0.264), T2 (p = 0.322) and T3 (p = 0.623). The percent women consuming omega-3 fatty acid rich foods in both NBW and LBW groups are shown in S1 Table.

## Discussion

### Main Findings

To the best of our knowledge this is the first report which has examined the levels of plasma as well as erythrocyte LCPUFA levels at 3 different time points during pregnancy and examined their association with cord blood LCPUFA levels in mothers delivering LBW babies. Our results indicate the following 1) Maternal erythrocyte DHA at T1 was positively associated with baby weight 2) Cord plasma and erythrocyte DHA levels were positively associated with maternal DHA levels at all the time points across the gestation 3) Higher maternal erythrocyte total omega-6 fatty acids and ARA levels at 26^th^-30^th^ week of pregnancy in women delivering LBW babies 4) Lower maternal erythrocyte omega-3 fatty acid and higher omega-6 fatty acid, SFA levels at delivery in women delivering LBW babies.

In the current study, maternal erythrocyte DHA levels at T1 were positively associated with birth weight, suggesting the potential benefits of DHA in influencing birth weight. This is supported by earlier studies which indicate the beneficial effects of DHA supplementation during pregnancy in increasing birth size [29, 30]. Studies have reported the positive association between the plasma DHA levels early in a normotensive pregnancy with birth weight [6, 17, 19]. Further, it is also suggested that dietary adaptation to adequate maternal fatty acid status helps in preventing fetal growth restriction which may improve health in later life [17]. This may have implications in improving neurodevelopment of the offspring. In addition, there was also a positive association of the both erythrocyte and plasma cord DHA levels with the maternal DHA levels right from the early pregnancy. However, in contrast, a series of meta-analysis recently reports that omega-3 fatty acid supplementation during pregnancy does not prevent recurrent preterm birth in asymptomatic singleton gestations with prior preterm birth; recurrent IUGR in asymptomatic singletons with prior IUGR; and do not reduce the incidence of preterm birth or improve neonatal outcome [31, 32, 33]. Further, omega-3 fatty acid supplementation during pregnancy was not associated with prevention of preterm birth, pre-eclampsia, IUGR, gestational diabetes, small for gestational age, post-partum depression or better children development [34].

We observed the negative association of the cord ARA levels with maternal ARA levels at T1 as well as at the time of delivery. These results are similar to our earlier reported study in normotensive pregnancy [28]. One possible explanation may be that the fetus is less dependent on the maternal ARA supply as compared to the DHA [35].

In the current study, maternal erythrocyte omega-6 fatty acid levels and ARA levels were higher at T2 as well as at delivery in mothers delivering LBW babies. Our findings are similar to studies in Amsterdam Born Children and their Development cohort, where they observed the association between higher levels of omega-6 LC-PUFA ARA and reduced birth weight in early pregnancy [6,17]. Similarly, other study in Maastricht Essential Fatty Acid Birth cohort also report a negative association between higher ARA levels during late pregnancy and at the time of delivery with decreased birth weight [19].

In the current study, we observed lower levels of maternal erythrocyte total omega-3 fatty acids at delivery in women delivering LBW babies. Importance of omega-3 fatty acid in fetal development during pregnancy is well established [36, 37]. A study carried out on south Indian subjects reports the association between the lower intake of fish during the third trimester of pregnancy and higher risk of delivering LBW babies [18]. During the third trimester of pregnancy, there is greatest accretion of LCPUFA by the fetus [38]. Omega-3 fatty acids are known to improve membrane fluidity and increase flow mediated vasodilation, thereby improving the membrane receptivity for various biologically active ligands. This may further lead to reduction in the blood viscosity and increase in the placental blood flow, thereby improving fetal growth [39]. Thus, the lower levels of omega-3 fatty acids might reflect the mother’s inability to supply adequate amounts of LCPUFA for optimal fetal development.

In the current study, we observed higher levels of maternal erythrocyte SFA levels at delivery in women delivering low birth weight babies. Besides, the importance of the LCPUFAs, the imbalanced maternal levels of saturated fatty acids may also have adverse effects on the developing fetus [40]. The higher levels of saturated fatty acids in women delivering LBW babies can possibly be attributed to the inadequate transfer of these fatty acids through placenta, contributing to inadequate fetal growth.

In the current study, the cord fatty acid levels i.e. DHA as well as ARA were higher as compared to maternal levels in both NBW and LBW group. There are several mechanisms in the placenta involving the action of lipases and fatty acid binding proteins for the preferential transfer of the critical LCPUFA to the fetal circulation [41]. This phenomenon is well known as biomagnification, where the fetus increases the LCPUFA percentage in fetal blood in order to support central nervous system development [42].

There could be several possible mechanisms leading to altered levels of LCPUFA in mothers delivering LBW babies as compared with mothers delivering NBW babies, despite of the observed similarity between the dietary intakes. 1) It is well known that enzymes like desaturases synthesize the omega-3 and omega-6 LCPUFA from their essential shorter chain precursors [37]. Thus, any alteration in the levels of these enzymes can affect the levels of fatty acids in the mother’s circulation. 2) There may be genetic variations from the single nucleotide polymorphism of the FADS1 and FADS2, which may influence the maternal plasma and erythrocyte phospholipid levels of omega-6 and omega-3 fatty acids during pregnancy [43]. 3) Our earlier study in normotensive pregnancy reports a negative association between maternal homocysteine and maternal DHA as well as omega-3 fatty acid levels at the time of delivery [28]. It is therefore likely that the levels of maternal micronutrients like folic acid and vitamin B_12_ may also influence the levels of maternal LCPUFA.

### Strengths and Limitations

The current study has some strengths as well as limitations. The strength of the study includes the measurement of dietary intake, plasma and erythrocyte fatty acid levels across the gestation and in the cord samples. Furthermore, the study is carried out in a homogenous population with women well matched for race and lifestyle patterns with no smoking, drug or alcohol use thereby reducing the effects of confounds. However, this study was non-randomized. Further, it also needs to be confirmed on a larger sample size. Further studies need to elucidate the mechanisms through which fatty acids influence birth size.

## Conclusion

Our data demonstrates a positive association of maternal DHA in early pregnancy with birth weight and therefore, suggests that supplementation of DHA may be useful in improving pregnancy outcome. There is now a considerable body of evidence which suggests that the quality of the early life environment of the fetus affects future disease risk and the current study has implications for the same. This study demonstrates the possible role of LCPUFA in the etiology of LBW babies’ right from early pregnancy.

## Supporting Information

S1 Table Frequency of consumption of foods rich in omega-3 fatty acids at three time points during pregnancy. NBW–Normal birth weight; LBW–Low birth weight; n—Number of subjects; p—Significance; T1 = 16th–20th week; T2 = 26th–30th week; T3 = at delivery.
(DOCX)
