# Supplementary material for: Maternal Fatty Acids and Their Association with Birth Outcome: A Prospective Study
Source: PLoS One. 2016 Jan 27;11(1):e0147359. doi: 10.1371/journal.pone.0147359 (PMC4729437; doi:10.1371/journal.pone.0147359)
Supplement: S1 Table — NBW–Normal birth weight; LBW–Low birth weight; n—Number of subjects; p—Significance; T1 = 16th–20th week; T2 = 26th–30th week; T3 = at delivery. (DOCX) [file pone.0147359.s001.docx]

**Table S1: Frequency of consumption of foods rich in omega-3 fatty acids at three time points during pregnancy.**

|  | **T1** | | | **T2** | | | **T3** | | |
| --- | --- | --- | --- | --- | --- | --- | --- | --- | --- |
| **Food Group**  **n (%)** | **NBW**  (n=51) | **LBW**  (n=44) | **p** | **NBW**  (n=30) | **LBW**  (n=37) | **p** | **NBW**  (n=46) | **LBW**  (n=40) | **p** |
| **Omega-3 Fatty Acid Rich Foods** | | | | | | | | | |
| Weekly twice | 16(31.4) | 21(47.7) | 0.103 | 10(33.3) | 19(51.4) | 0.139 | 13(28.3) | 15(37.5) | 0.362 |
| Weekly 3-6 times | 24(47.1) | 16(36.4) | 0.292 | 12(40.0) | 10(27.0) | 0.261 | 17(37.0) | 14(35.0) | 0.851 |
| Weekly more than 6 times | 11(21.6) | 7(15.9) | 0.483 | 8(26.7) | 8(21.6) | 0.630 | 16(34.8) | 11(27.5) | 0.468 |

^NBW– Normal birth weight; LBW–Low birth weight; n - Number of subjects; p - Significance; T1=16th - 20th week; T2=26th - 30th week; T3= at delivery.^
